# Crystal structure of 3-(benzo[*d*]thia­zol-2-yl)-6-methyl-2*H*-chromen-2-one

**DOI:** 10.1107/S205698902300347X

**Published:** 2023-04-25

**Authors:** Amira E. M. Abdallah, Galal H. Elgemeie, Peter G. Jones

**Affiliations:** a Chemistry Department, Faculty of Science, Helwan University, Cairo, Egypt; b Institut für Anorganische und Analytische Chemie, Technische Universität Braunschweig, Hagenring 30, D-38106 Braunschweig, Germany; Universität Greifswald, Germany

**Keywords:** benzo­thia­zole, coumarin, crystal structure

## Abstract

The mol­ecule of the title compound, C_17_H_11_NO_2_S, is almost planar, with an inter­planar angle of 3.01 (3)° between the benzo­thia­zole and chromene ring systems. A short intra­molecular S⋯O=C contact of 2.727 (2) Å is observed. The crystal packing involves a layer structure parallel to (211), containing dimeric inversion-symmetric units connected by a ‘weak’ C—H⋯O=C hydrogen bond.

## Abstract 2

The title mol­ecule is almost planar, with an intra­molecular S⋯O=C contact. The packing is a layer structure with dimeric units connected by a C—H⋯O=C hydrogen bond.

## 1. Chemical context

Benzo­thia­zole and its derivatives are important heterocyclic aromatic compounds. Benzo­thia­zole can be readily substituted at the C-2 position of the thia­zole ring (Elgemeie *et al.*, 2020 ▸). Compounds containing a benzo­thia­zolyl group have found numerous applications in medicine and in nonlinear optics (Sigmundová *et al.*, 2007 ▸). Benzo­thia­zole derivatives can also display strong fluorescence and luminescence in the solid state and in solution (Wang *et al.*, 2010 ▸). Benzo­thia­zole compounds as incorporated in organic light-emitting diodes have attracted substantial attention because of their notable photovoltaic properties (Ghanavatkar *et al.*, 2020 ▸). Recently, we have synthesized novel heterocyclic derivatives involving the benzo­thia­zole moiety, many of which showed significant biological and fluorescence activities (Azzam *et al.*, 2020 ▸; Khedr *et al.*, 2022 ▸). 
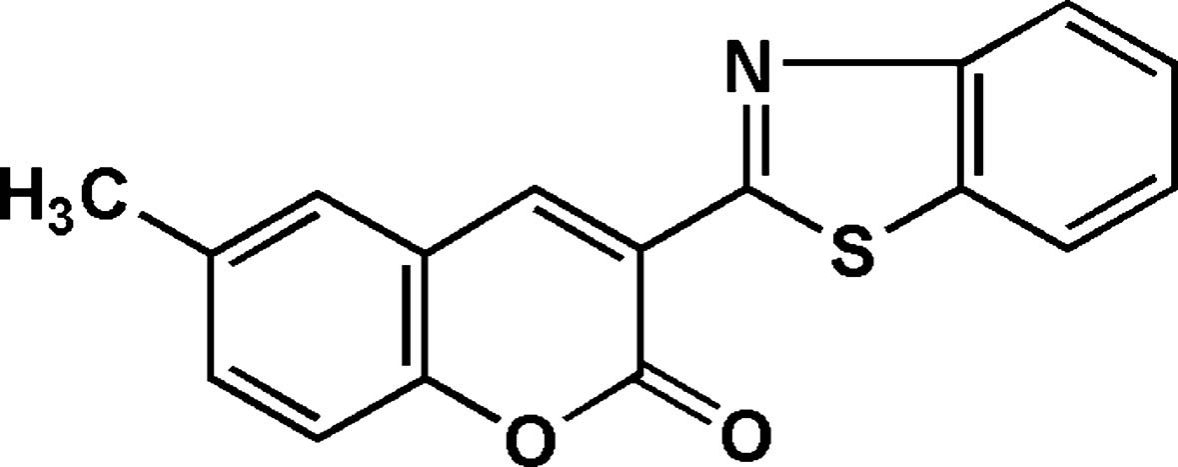


Coumarin is a natural product with the systematic name 2*H*-chromen-2-one. Its derivatives represent an important class of organic heterocycles. Thus, they are constituents of many intensively investigated and commercially important organic fluorescent materials; they also display important biological activities and are found in synthetic drugs (Curini *et al.*, 2006 ▸). Furthermore, many coumarin compounds are current photosensitizers with valuable applications in medicinal chemistry (Bansal *et al.*, 2013 ▸). Because of their photochemical properties, coumarin compounds have found applications in nonlinear optical materials, solar energy collectors and charge-transfer agents (Kim *et al.*, 2011 ▸), and also as daylight fluorescent pigments, tunable dye lasers, fluorescent probes and components of organic light-emitting diodes (Christie & Lui, 2000 ▸). The emission intensities of coumarin chromophores depend on the nature of their substituents at various sites (Żamojć *et al.*, 2014 ▸).

In two recent papers (Abdallah *et al.*, 2022 ▸, 2023 ▸), we have given a more extensive list of references concerning the pro­perties of benzo­thia­zoles and coumarins, including many of our own publications in these fields.

Some decades ago, we reported the syntheses and characterizations of novel coumarin derivatives that have found applications as laser dyes (Elgemeie, 1989 ▸); these included 3-(benzo[*d*]thia­zol-2-yl)-2*H*-chromen-2-one, the desmethyl ana­logue of title compound **4**, a benzo­thia­zole-based cou­marin derivative which was synthesized by the reaction of 2-(cyano­meth­yl)benzo­thia­zole with salicyaldehyde. Afterwards, other research groups utilized essentially the same reaction to synthesize different derivatives of the same heterocyclic framework, including compound **4** (Chao *et al.*, 2010 ▸; Makowska *et al.*, 2019 ▸).

**Figure 1:**
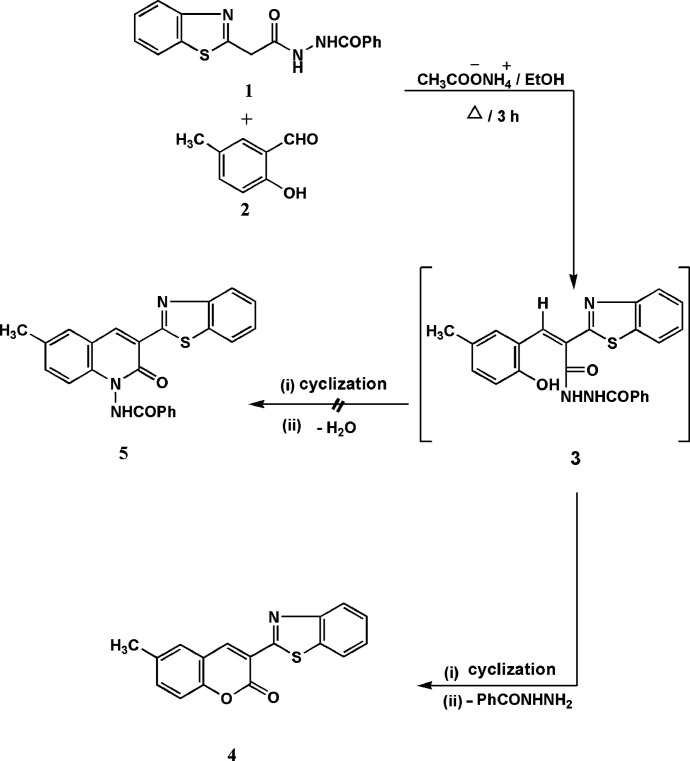
Reaction scheme showing the the attempted synthesis of compound **5**, which led instead to the product **4**.

Recently, we attempted to synthesize *N*-[3-(benzo[*d*]thia­zol-2-yl)-6-methyl-2-oxoquinolin-1(2*H*)-yl]benzamide (**5**) by the reaction of *N*-[2-(benzo[*d*]thia­zol-2-yl)acet­yl]benzo­hy­dra­zide (**1**) (Azzam *et al.*, 2021 ▸) with 5-methyl­salicylaldehyde (**2**) (Fig. 1 ▸). However, the product gave a mass spectrum that was inconsistent with the proposed structure. Therefore, the X-ray crystal structure was determined, indicating the form­ation of 3-(benzo[*d*]thia­zol-2-yl)-6-methyl-2*H*-chro­men-2-one (**4**) as the sole product, presumably arising *via* the initial form­ation of adduct **3** and the subsequent elimination of benzohydrazide rather than water. This is consistent with our recent observation that the desmethyl compound mentioned above is also formed as an unintended product by an exactly analogous reaction (Abdallah *et al.*, 2022 ▸). The products thus represent, by coincidence, a continuation of our research on developing new benzo­thia­zole and coumarin derivatives as organic fluorescent constituents (Elgemeie *et al.*, 2015 ▸).

## 2. Structural commentary

**Figure 2:**
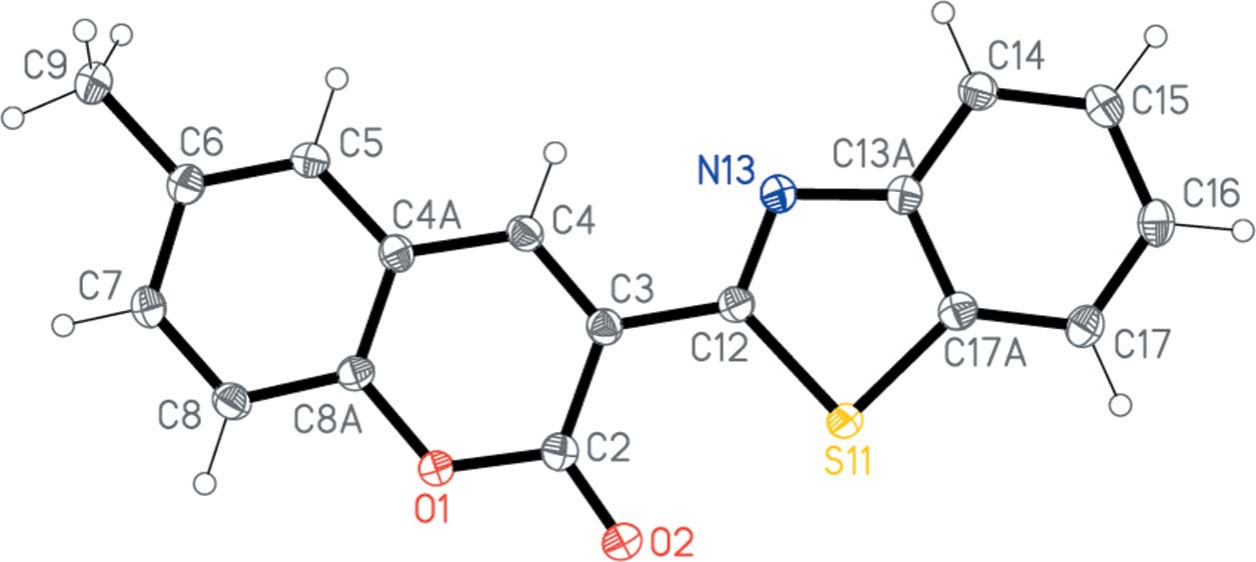
The mol­ecule of compound **4** in the crystal. Displacement ellipsoids represent 50% probability levels.

The structure of compound **4** is shown in Fig. 2 ▸. Its bond lengths and angles may be regarded as normal; a selection is presented in Table 1 ▸. The chromene and benzo­thia­zole ring systems are planar as expected, with respective r.m.s. deviations of 0.020 and 0.015 Å; the angle between these planes is only 3.01 (3)°, so that the whole mol­ecule almost planar. A short intra­molecular S11⋯O2 contact of 2.792 (1) Å is observed. The desmethyl analogue (Abdallah *et al.*, 2022 ▸) has, as expected, a closely similar mol­ecular structure, with an S⋯O=C contact of 2.727 (2) Å and an inter­planar angle of 6.47 (6)°, but is not isotypic to **4**.

## 3. Supra­molecular features

**Figure 3:**
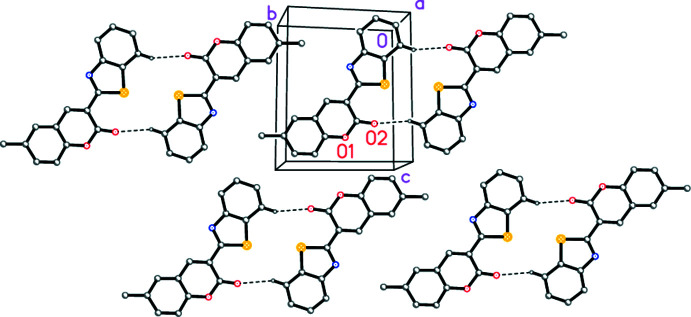
Packing diagram of compound **4**, viewed perpendicular to (211). Dashed lines indicate ‘weak’ hydrogen bonds. Atom labels indicate the asymmetric unit. H atoms other than H17 have been omitted.

The short contact H17⋯O2 (at −*x* + 2, −*y*, −*z* + 1; see Table 2 ▸) may be regarded as a ‘weak’ hydrogen bond. It links the mol­ecules to form inversion-symmetric dimers (Fig. 3 ▸) in which the S⋯S distance is 3.678 (1) Å. Adjacent dimers are related by translation to give an extended structure consisting of layers parallel to (211). The inter­centroid distances (in Å) between rings of neighbouring layers, defining rings *A*–*D* as thia­zole, the arene ring of benzo­thia­zole, pyran and the arene ring of chromene, respectively, are *A*⋯*C* = 3.64, *A*⋯*D* = 3.70 and *B*⋯*D* = 3.61 [all with the symmetry code (−*x* + 1, −*y* + 1, −*z* + 1)], *B*⋯*C* = 3.51 and *B*⋯*D* = 3.66 Å [both with the symmetry code (−*x* + 2, −*y* + 1, −*z* + 1)]. The ring offsets (in the same order) are 1.44, 1.32, 0.80, 0.86 and 1.39 Å.

## 4. Database survey

The searches employed the routine *ConQuest* (Bruno *et al.*, 2002 ▸), part of Version 2022.3.0 of the Cambridge Structural Database (Groom *et al.*, 2016 ▸). A search for structures con­taining both a coumarin and a benzo­thia­zole ring system in the same residue gave 16 hits. After excluding ring systems with more extended annelation and mol­ecules where the ring systems were not directly bonded to each other, 7 hits remained. In all of these, the benzo­thia­zole was bonded *via* its 2-position. The structure with refcode AKUCUG (Bakthadoss & Selvakumar, 2016 ▸) involved a linkage *via* the 8-position of the coumarin; the other 6 hits [VIVWEF and VIWDOX (Shi *et al.*, 2019 ▸); WINZAU (Jasinski & Paight, 1995 ▸); SECSEC (Abdallah *et al.*, 2022 ▸); PEGMEX and PEGMIB (Singh *et al.*, 2022 ▸)] all had this linkage at the 3-position, as in compound **4**. In all cases, a short intra­molecular S⋯O=C contact was observed, with distances in the range 2.681–2.786 Å.

## 5. Synthesis and crystallization

5-Methyl­salicyl­aldehyde, **2** (1.36 g, 0.01 mol) and ammonium acetate (0.77 g, 0.01 mol) were added to a solution of *N*-[2-(benzo[*d*]thia­zol-2-yl)acet­yl]benzohydrazide, **1** (3.11 g, 0.01 mol), in ethanol (10 mL). The reaction mixture was refluxed for *ca* 3 h and the resulting precipitate was collected by filtration and recrystallized from ethanol.

Pale-yellow crystals; yield: 96% (2.82 g); m.p. 495–497 K; IR (KBr, cm^−1^): ν 3062, (CH-aromatic), 2918 (CH_3_), 1710 (C=O), 1582 (C=N) and 1619, 1485 (C=C). ^1^H NMR (400 MHz, DMSO-*d*_6_): δ 2.40 (*s*, 3H, CH_3_), 7.42–8.18 (*m*, 7H, C_6_H_4_, C_6_H_3_), 9.19 (*s*, 1H, CH-pyran). ^13^C NMR (100 MHz, DMSO-*d_6_*): δ 20.8 (CH_3_), 116.5, 119.0, 120.0, 122.7, 123.0, 124.5, 125.9, 127.2, 128.6, 130.2, 135.2, 136.4, 142.4, 152.4 (aromatic C atoms, pyran ring), 160.0 (C=N), 160.4 (C=O). MS (EI): *m*/*z* (%) 293 [*M* ^+^] (100.00). Analysis calculated (%) for C_17_H_11_NO_2_S: C 69.61, H 3.78, N 4.77, S 10.93; found: C 69.42, H 3.90, N 4.66, S 10.99.

## 6. Refinement

The title crystal was a non-merohedral two-component twin. The orientations are related by a 180° rotation around the reciprocal axis c*. The structure was refined using the HKLF5 method, whereby the relative volume of the smaller twin component refined to 0.387 (1). For non-merohedral twins thus refined, *R*_int_ is not a valid concept, and the number of reflections should be inter­preted with caution, because the equivalent reflections in the intensity file have already been merged.

Crystal data, data collection and structure refinement details are summarized in Table 3 ▸. The methyl group was included as an idealized rigid group allowed to rotate but not tip (C—H = 0.98 Å and H—C—H = 109.5°). Other H atoms were included using a riding model starting from calculated positions (aromatic C—H = 0.95 Å). The *U*_iso_(H) values were fixed at 1.5 times *U*_eq_ of the parent C atoms for methyl groups and at 1.2 times *U*_eq_ for the other H atoms.

## Supplementary Material

Crystal structure: contains datablock(s) I, global. DOI: 10.1107/S205698902300347X/yz2033sup1.cif

Structure factors: contains datablock(s) I. DOI: 10.1107/S205698902300347X/yz2033Isup2.hkl

CCDC reference: 2256779

Additional supporting information:  crystallographic information; 3D view; checkCIF report

## Figures and Tables

**Table 1:** Selected geometric parameters (Å, °)

O1—C2	1.3723 (17)	S11—C17*A*	1.7343 (14)
O1—C8*A*	1.3765 (18)	S11—C12	1.7515 (15)
O2—C2	1.2077 (19)	C12—N13	1.3076 (18)
C3—C12	1.4698 (19)	N13—C13*A*	1.3836 (18)
			
C2—O1—C8*A*	122.52 (11)	N13—C12—S11	115.94 (11)
O2—C2—O1	116.99 (12)	C3—C12—S11	123.35 (10)
O2—C2—C3	125.69 (14)	N13—C13*A*—C14	124.90 (13)
O1—C2—C3	117.32 (13)	N13—C13*A*—C17*A*	115.23 (12)
C17*A*—S11—C12	88.94 (7)	C17—C17*A*—S11	129.05 (12)
N13—C12—C3	120.72 (13)	C13*A*—C17*A*—S11	109.42 (11)

**Table 2:** Hydrogen-bond geometry (Å, °)

*D*—H⋯*A*	*D*—H	H⋯*A*	*D*⋯*A*	*D*—H⋯*A*
C17—H17⋯O2^i^	0.95	2.43	3.3235 (19)	157

Symmetry code: (i) 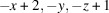 .

**Table 3:** Experimental details

Crystal data	
Chemical formula	C_17_H_11_NO_2_S
*M*_r_	293.33
Crystal system, space group	Triclinic, *P* 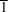
Temperature (K)	100
*a*, *b*, *c* (Å)	7.1592 (2), 9.0048 (2), 10.8678 (3)
α, β, γ (°)	82.779 (2), 76.016 (2), 75.078 (2)
*V* (Å^3^)	655.42 (3)
*Z*	2
Radiation type	Cu *K*α
μ (mm^−1^)	2.22
Crystal size (mm)	0.2 × 0.08 × 0.03

Data collection	
Diffractometer	Rigaku XtaLAB Synergy
Absorption correction	Multi-scan (*CrysAlis PRO*; Rigaku OD, 2022 ▸)
*T*_min_, *T*_max_	0.391, 1.000
No. of measured, independent and observed [*I* > 2σ(*I*)] reflections	4408, 4408, 4313
*R*_int_	See *Refinement*
(sin θ/λ)_max_ (Å^−1^)	0.634

Refinement	
*R*[*F* ^2^ > 2σ(*F* ^2^)], *wR*(*F* ^2^), *S*	0.035, 0.102, 1.07
No. of reflections	4408
No. of parameters	192
H-atom treatment	H-atom parameters constrained
Δρ_max_, Δρ_min_ (e Å^−3^)	0.36, −0.28

Computer programs: *CrysAlis PRO* (Rigaku OD, 2022 ▸), *SHELXT* (Sheldrick, 2015*a* ▸), *SHELXL2018* (Sheldrick, 2015*b* ▸) and *XP* (Siemens, 1994 ▸).

## supplementary crystallographic information

### Crystal data

**Table:**

C_17_H_11_NO_2_S	*Z* = 2
*M**_r_* = 293.33	*F*(000) = 304
Triclinic, *P*1	*D*_x_ = 1.486 Mg m^−^^3^
*a* = 7.1592 (2) Å	Cu *K*α radiation, λ = 1.54184 Å
*b* = 9.0048 (2) Å	Cell parameters from 15669 reflections
*c* = 10.8678 (3) Å	θ = 5.1–76.9°
α = 82.779 (2)°	µ = 2.22 mm^−^^1^
β = 76.016 (2)°	*T* = 100 K
γ = 75.078 (2)°	Lath, yellow
*V* = 655.42 (3) Å^3^	0.2 × 0.08 × 0.03 mm

### Data collection

**Table:**

Rigaku XtaLAB Synergy diffractometer	4408 measured reflections
Radiation source: micro-focus sealed X-ray tube, PhotonJet (Cu) X-ray Source	4408 independent reflections
Mirror monochromator	4313 reflections with *I* > 2σ(*I*)
Detector resolution: 10.0000 pixels mm^-1^	θ_max_ = 77.7°, θ_min_ = 4.2°
ω scans	*h* = −9→9
Absorption correction: multi-scan (CrysAlis PRO; Rigaku OD, 2022)	*k* = −11→11
*T*_min_ = 0.391, *T*_max_ = 1.000	*l* = −13→13

### Refinement

**Table:**

Refinement on *F*^2^	Primary atom site location: dual
Least-squares matrix: full	Hydrogen site location: inferred from neighbouring sites
*R*[*F*^2^ > 2σ(*F*^2^)] = 0.035	H-atom parameters constrained
*wR*(*F*^2^) = 0.102	*w* = 1/[σ^2^(*F*_o_^2^) + (0.069*P*)^2^ + 0.1477*P*] where *P* = (*F*_o_^2^ + 2*F*_c_^2^)/3
*S* = 1.07	(Δ/σ)_max_ = 0.001
4408 reflections	Δρ_max_ = 0.36 e Å^−^^3^
192 parameters	Δρ_min_ = −0.28 e Å^−^^3^
0 restraints	

### Special details

**Table:**

Geometry. All esds (except the esd in the dihedral angle between two l.s. planes) are estimated using the full covariance matrix. The cell esds are taken into account individually in the estimation of esds in distances, angles and torsion angles; correlations between esds in cell parameters are only used when they are defined by crystal symmetry. An approximate (isotropic) treatment of cell esds is used for estimating esds involving l.s. planes.

### Fractional atomic coordinates and isotropic or equivalent isotropic displacement parameters (Å^2^)

**Table:**

	*x*	*y*	*z*	*U*_iso_*/*U*_eq_	
O1	0.51103 (16)	0.48246 (12)	0.81168 (9)	0.0190 (2)	
O2	0.6896 (2)	0.26471 (13)	0.73105 (10)	0.0284 (3)	
C2	0.6325 (2)	0.40210 (18)	0.71141 (13)	0.0193 (3)	
C3	0.6808 (2)	0.48993 (17)	0.58998 (12)	0.0165 (3)	
C4	0.6030 (2)	0.64361 (17)	0.57955 (12)	0.0167 (3)	
H4	0.632732	0.699040	0.499800	0.020*	
C4A	0.4771 (2)	0.72457 (17)	0.68581 (13)	0.0163 (3)	
C5	0.3967 (2)	0.88415 (17)	0.68151 (13)	0.0180 (3)	
H5	0.421660	0.943350	0.603163	0.022*	
C6	0.2821 (2)	0.95747 (18)	0.78852 (13)	0.0186 (3)	
C7	0.2475 (2)	0.86682 (17)	0.90335 (13)	0.0179 (3)	
H7	0.169855	0.915469	0.977831	0.022*	
C8A	0.4369 (2)	0.63944 (17)	0.80150 (13)	0.0163 (3)	
C8	0.3231 (2)	0.70939 (17)	0.91095 (13)	0.0185 (3)	
H8	0.297826	0.650194	0.989268	0.022*	
C9	0.1992 (3)	1.12900 (19)	0.78301 (15)	0.0253 (3)	
H9A	0.109385	1.158236	0.864314	0.038*	
H9B	0.126469	1.159123	0.714667	0.038*	
H9C	0.307944	1.181373	0.766476	0.038*	
S11	0.92276 (5)	0.21156 (4)	0.48745 (3)	0.01826 (13)	
C12	0.8130 (2)	0.40930 (16)	0.48039 (13)	0.0163 (3)	
N13	0.85618 (18)	0.48415 (14)	0.37018 (11)	0.0170 (3)	
C13A	0.9840 (2)	0.38535 (17)	0.28225 (13)	0.0167 (3)	
C14	1.0636 (2)	0.42978 (18)	0.15610 (13)	0.0189 (3)	
H14	1.029684	0.533983	0.123985	0.023*	
C15	1.1927 (2)	0.31842 (18)	0.07949 (13)	0.0200 (3)	
H15	1.249720	0.347072	−0.005730	0.024*	
C16	1.2408 (2)	0.16354 (18)	0.12571 (14)	0.0205 (3)	
H16	1.327948	0.089102	0.070564	0.025*	
C17A	1.0364 (2)	0.23021 (17)	0.32803 (13)	0.0177 (3)	
C17	1.1641 (2)	0.11734 (18)	0.24965 (14)	0.0198 (3)	
H17	1.196910	0.012568	0.280584	0.024*	

### Atomic displacement parameters (Å^2^)

**Table:**

	*U*^11^	*U*^22^	*U*^33^	*U*^12^	*U*^13^	*U*^23^
O1	0.0238 (6)	0.0148 (5)	0.0158 (5)	−0.0030 (4)	−0.0012 (4)	−0.0006 (4)
O2	0.0399 (7)	0.0162 (6)	0.0212 (5)	−0.0006 (5)	0.0006 (5)	0.0007 (4)
C2	0.0228 (7)	0.0171 (7)	0.0174 (6)	−0.0044 (6)	−0.0029 (5)	−0.0018 (5)
C3	0.0178 (7)	0.0169 (7)	0.0151 (6)	−0.0046 (5)	−0.0036 (5)	−0.0016 (5)
C4	0.0188 (7)	0.0177 (7)	0.0141 (6)	−0.0050 (6)	−0.0036 (5)	−0.0008 (5)
C4A	0.0165 (7)	0.0178 (7)	0.0153 (6)	−0.0044 (6)	−0.0037 (5)	−0.0021 (5)
C5	0.0196 (7)	0.0173 (7)	0.0163 (6)	−0.0043 (6)	−0.0034 (5)	0.0009 (5)
C6	0.0179 (7)	0.0181 (7)	0.0198 (6)	−0.0034 (6)	−0.0046 (5)	−0.0024 (5)
C7	0.0157 (7)	0.0205 (7)	0.0167 (6)	−0.0029 (5)	−0.0017 (5)	−0.0041 (5)
C8A	0.0170 (7)	0.0149 (7)	0.0174 (6)	−0.0047 (5)	−0.0039 (5)	−0.0004 (5)
C8	0.0198 (7)	0.0197 (7)	0.0158 (6)	−0.0060 (6)	−0.0024 (5)	0.0002 (5)
C9	0.0317 (8)	0.0180 (8)	0.0217 (7)	−0.0002 (6)	−0.0027 (6)	−0.0034 (5)
S11	0.0232 (2)	0.01407 (19)	0.01565 (19)	−0.00208 (14)	−0.00330 (13)	−0.00092 (13)
C12	0.0172 (6)	0.0160 (7)	0.0162 (6)	−0.0040 (5)	−0.0046 (5)	−0.0011 (5)
N13	0.0174 (6)	0.0172 (6)	0.0160 (5)	−0.0038 (5)	−0.0031 (4)	−0.0018 (4)
C13A	0.0157 (7)	0.0168 (7)	0.0180 (6)	−0.0038 (5)	−0.0037 (5)	−0.0027 (5)
C14	0.0190 (7)	0.0190 (7)	0.0184 (6)	−0.0047 (6)	−0.0038 (5)	−0.0003 (5)
C15	0.0186 (7)	0.0240 (8)	0.0175 (6)	−0.0067 (6)	−0.0017 (5)	−0.0028 (5)
C16	0.0185 (7)	0.0227 (8)	0.0211 (7)	−0.0041 (6)	−0.0036 (5)	−0.0076 (6)
C17A	0.0186 (7)	0.0171 (7)	0.0186 (6)	−0.0052 (6)	−0.0051 (5)	−0.0016 (5)
C17	0.0206 (7)	0.0176 (7)	0.0216 (7)	−0.0036 (6)	−0.0054 (5)	−0.0039 (5)

### Geometric parameters (Å, º)

**Table:**

O1—C2	1.3723 (17)	C13A—C14	1.4022 (19)
O1—C8A	1.3765 (18)	C13A—C17A	1.409 (2)
O2—C2	1.2077 (19)	C14—C15	1.383 (2)
C2—C3	1.4645 (19)	C15—C16	1.406 (2)
C3—C4	1.354 (2)	C16—C17	1.381 (2)
C3—C12	1.4698 (19)	C17A—C17	1.399 (2)
C4—C4A	1.4310 (19)	C4—H4	0.9500
C4A—C8A	1.3956 (19)	C5—H5	0.9500
C4A—C5	1.403 (2)	C7—H7	0.9500
C5—C6	1.384 (2)	C8—H8	0.9500
C6—C7	1.4083 (19)	C9—H9A	0.9800
C6—C9	1.504 (2)	C9—H9B	0.9800
C7—C8	1.381 (2)	C9—H9C	0.9800
C8A—C8	1.3897 (19)	C14—H14	0.9500
S11—C17A	1.7343 (14)	C15—H15	0.9500
S11—C12	1.7515 (15)	C16—H16	0.9500
C12—N13	1.3076 (18)	C17—H17	0.9500
N13—C13A	1.3836 (18)		
			
C2—O1—C8A	122.52 (11)	C14—C15—C16	121.07 (13)
O2—C2—O1	116.99 (12)	C17—C16—C15	121.37 (14)
O2—C2—C3	125.69 (14)	C17—C17A—C13A	121.53 (13)
O1—C2—C3	117.32 (13)	C17—C17A—S11	129.05 (12)
C4—C3—C2	119.91 (13)	C13A—C17A—S11	109.42 (11)
C4—C3—C12	120.78 (12)	C16—C17—C17A	117.68 (14)
C2—C3—C12	119.31 (13)	C3—C4—H4	119.2
C3—C4—C4A	121.57 (12)	C4A—C4—H4	119.2
C8A—C4A—C5	118.28 (13)	C6—C5—H5	119.2
C8A—C4A—C4	117.62 (13)	C4A—C5—H5	119.2
C5—C4A—C4	124.07 (12)	C8—C7—H7	119.0
C6—C5—C4A	121.65 (13)	C6—C7—H7	119.0
C5—C6—C7	117.94 (14)	C7—C8—H8	120.7
C5—C6—C9	121.11 (13)	C8A—C8—H8	120.7
C7—C6—C9	120.94 (13)	C6—C9—H9A	109.5
C8—C7—C6	122.00 (13)	C6—C9—H9B	109.5
O1—C8A—C8	117.40 (12)	H9A—C9—H9B	109.5
O1—C8A—C4A	121.01 (13)	C6—C9—H9C	109.5
C8—C8A—C4A	121.59 (14)	H9A—C9—H9C	109.5
C7—C8—C8A	118.53 (13)	H9B—C9—H9C	109.5
C17A—S11—C12	88.94 (7)	C15—C14—H14	120.8
N13—C12—C3	120.72 (13)	C13A—C14—H14	120.8
N13—C12—S11	115.94 (11)	C14—C15—H15	119.5
C3—C12—S11	123.35 (10)	C16—C15—H15	119.5
C12—N13—C13A	110.47 (12)	C17—C16—H16	119.3
N13—C13A—C14	124.90 (13)	C15—C16—H16	119.3
N13—C13A—C17A	115.23 (12)	C16—C17—H17	121.2
C14—C13A—C17A	119.86 (13)	C17A—C17—H17	121.2
C15—C14—C13A	118.48 (14)		
			
C8A—O1—C2—O2	179.74 (13)	C4A—C8A—C8—C7	0.5 (2)
C8A—O1—C2—C3	−0.6 (2)	C4—C3—C12—N13	−0.9 (2)
O2—C2—C3—C4	178.35 (15)	C2—C3—C12—N13	178.82 (13)
O1—C2—C3—C4	−1.3 (2)	C4—C3—C12—S11	178.79 (11)
O2—C2—C3—C12	−1.3 (2)	C2—C3—C12—S11	−1.53 (19)
O1—C2—C3—C12	178.98 (12)	C17A—S11—C12—N13	−0.22 (11)
C2—C3—C4—C4A	1.5 (2)	C17A—S11—C12—C3	−179.88 (12)
C12—C3—C4—C4A	−178.79 (13)	C3—C12—N13—C13A	179.37 (12)
C3—C4—C4A—C8A	0.1 (2)	S11—C12—N13—C13A	−0.31 (15)
C3—C4—C4A—C5	178.18 (13)	C12—N13—C13A—C14	−178.01 (13)
C8A—C4A—C5—C6	0.7 (2)	C12—N13—C13A—C17A	0.85 (17)
C4—C4A—C5—C6	−177.38 (13)	N13—C13A—C14—C15	178.86 (13)
C4A—C5—C6—C7	0.0 (2)	C17A—C13A—C14—C15	0.1 (2)
C4A—C5—C6—C9	179.11 (13)	C13A—C14—C15—C16	1.1 (2)
C5—C6—C7—C8	−0.4 (2)	C14—C15—C16—C17	−1.2 (2)
C9—C6—C7—C8	−179.54 (14)	N13—C13A—C17A—C17	179.81 (13)
C2—O1—C8A—C8	−177.01 (13)	C14—C13A—C17A—C17	−1.3 (2)
C2—O1—C8A—C4A	2.3 (2)	N13—C13A—C17A—S11	−1.00 (16)
C5—C4A—C8A—O1	179.81 (12)	C14—C13A—C17A—S11	177.92 (11)
C4—C4A—C8A—O1	−2.0 (2)	C12—S11—C17A—C17	179.76 (15)
C5—C4A—C8A—C8	−1.0 (2)	C12—S11—C17A—C13A	0.66 (11)
C4—C4A—C8A—C8	177.23 (13)	C15—C16—C17—C17A	0.0 (2)
C6—C7—C8—C8A	0.2 (2)	C13A—C17A—C17—C16	1.2 (2)
O1—C8A—C8—C7	179.80 (12)	S11—C17A—C17—C16	−177.78 (11)

### Hydrogen-bond geometry (Å, º)

**Table:**

*D*—H···*A*	*D*—H	H···*A*	*D*···*A*	*D*—H···*A*
C17—H17···O2^i^	0.95	2.43	3.3235 (19)	157

Symmetry code: (i) −*x*+2, −*y*, −*z*+1.
